# Animal Welfare, Carcass-Processing Practices and Post-Mortem Lesions in Nigerian Municipal Slaughterhouses: Implications for Meat Quality and Public Health Security

**DOI:** 10.3390/vetsci13050439

**Published:** 2026-04-30

**Authors:** Emmanuel O. Njoga, Jameslove I. Kperegbeyi, Onyinye S. Onwumere-Idolor, Uzezi G. Imonikebe, Chidiebere O. Anyaoha, Lynda O. Majesty-Alukagberie, Joel C. Ugwunwarua, Nnaedozie E. Onah, James W. Oguttu

**Affiliations:** 1 Department of Agriculture and Animal Health, College of Agriculture and Environmental Sciences, Florida Science Campus, University of South Africa, Roodepoort 1709, Johannesburg, South Africa; joguttu@unisa.ac.za; 2 Department of Veterinary Public Health and Preventive Medicine, Faculty of Veterinary Medicine, University of Nigeria, Nsukka 410001, Enugu State, Nigeria; chidiebere.anyaoha@unn.edu.ng (C.O.A.); lynda.majesty-alukagberie@unn.edu.ng (L.O.M.-A.); johelconsults@gmail.com (J.C.U.); nnaedozieemmanue93@gmail.com (N.E.O.); 3 Department of Animal Production, Faculty of Agriculture, Southern Delta University, PMB 005, Ozoro 334111, Delta State, Nigeria; jameslovekperegbeyi2024@gmail.com (J.I.K.); onwumere-idoloros@dsust.edu.ng (O.S.O.-I.);

**Keywords:** disease surveillance, food safety, meat quality, public health, slaughterhouse hygiene

## Abstract

This five-month epidemiological investigation evaluated pre-slaughter welfare, carcass-processing practices, and post-mortem lesion prevalence in 1012 cattle and 413 pigs slaughtered in Enugu State, Nigeria. Direct observations and post-mortem inspections were conducted following OIE standards. Animal welfare was markedly compromised. Cattle were dragged from the lairage to kill floor, restrained in lateral recumbency for over 30 min before bleeding, and slaughtered without stunning. Pigs were transported tied to motorcycles and processed on unsanitary floors. The lairages lacked roofing, clean water, and adequate drainage. Carcass handling was unhygienic, with meat processed near maggot-infested drains and transported in open vans or motorized tricycles used to commute passengers and cement. Of all cattle examined, 45.3% (458/1012) exhibited gross lesions attributable to contagious bovine pleuropneumonia (CBPP, 15.5%), fasciolosis (18%), liver abscessation (6.6%), ascariasis (4.6%), and bovine tuberculosis (0.5%). No lesions were detected in pigs. Lesion occurrence differed significantly (*p* < 0.05) by sex (males = 44.1%, females = 66.7%), age (<4 years = 54.1%, ≥4 years = 45.4%), breed (White Fulani = 45.5%, others = 36.7%), slaughterhouse location, and season (rainy = 45.2%, dry = 45.5%). Temporal analysis showed the highest lesion rate in April (68.3%), declining to 37.7% in May. Lesions of CBPP and fasciolosis were significantly more frequent in young cattle and during the rainy months (*p* < 0.05). These findings reveal systemic welfare violations and disease endemicity within the municipal abattoirs surveyed. The combination of poor pre-slaughter welfare, unhygienic meat handling, and high prevalence of zoonotic and economically important livestock disease lesions highlights urgent public health concerns. Strengthening abattoir infrastructure, enforcing pre-slaughter animal welfare and hygiene regulations, mechanizing slaughter processes, and instituting continuous surveillance within the One Health framework are essential for ensuring meat safety and public health security in Nigeria and beyond.

## Simple Summary

This study evaluated the pre-slaughter animal welfare, carcass-processing practices, and prevalence of post-mortem lesions in selected Nigerian municipal slaughterhouses. The study revealed widespread inhumane animal handling, lack of pre-slaughter stunning, inadequate lairage facilities, and unhygienic carcass processing practices. Post-mortem examination showed that 45.3% of cattle harboured pathological lesions of zoonotic and economically important diseases, predominantly fasciolosis (18%), contagious bovine pleuropneumonia (15.5%), liver abscesses (6.6%), ascariasis (4.6%), and bovine tuberculosis (0.5%). No lesions were detected in pig carcasses inspected. Lesion prevalence varied significantly with sex, age, breed, season, and slaughterhouse location (*p* < 0.05), with the highest occurrence recorded in April. Additionally, poor meat handling practices, including processing on visibly unclean floors and transporting carcasses in open, non-specialized vehicles, were identified as critical risk factors for microbial contamination and zoonotic disease transmission. The findings highlight the endemicity of economically important livestock and zoonotic diseases, and underscore the urgent need to improve slaughterhouse infrastructure, enforce animal welfare compliance and hygiene standards in the study area, to ensure food safety and human health security.

## 1. Introduction

Meat remains central to human food culture globally. Slaughterhouses are vital for safeguarding animal welfare, ensuring meat safety, and enabling disease surveillance through ante- and post-mortem inspections. Pre-slaughter animal welfare, carcass handling, and post-mortem lesions remain critical determinants of meat quality, safety, and zoonotic risks, particularly in low- and middle-income countries [1]. In Nigeria, much of the meat consumed comes from municipal slaughter slabs and slaughterhouses, where animal welfare and hygiene standards are often compromised [1,2]. Poor pre-slaughter conditions such as transportation stress, inadequate holding lairage, and inhumane slaughter methods have been directly associated with animal suffering, immune suppression, and increased prevalence of pathological lesions, which may compromise meat quality and safety, and exacerbate the risk of zoonotic disease transmission [1,2,3,4]. Inadequate training and poor awareness on modes of zoonotic disease transmission among abattoir workers, coupled with poor enforcement of veterinary public health regulations, continue to enhance zoonotic disease transmission, particularly in developing economies where meat inspection and hygiene are suboptimal [5,6,7].

Nigeria is among African countries where a large share of meat consumed is processed in informal and semi-formal slaughterhouses, and this is largely due to clandestine slaughter and inadequate slaughterhouse operations [1,2]. Slaughter facilities in Enugu State, Nigeria, often operate under minimal regulatory oversight, with common practices including the use of rudimentary slaughtering tools, the absence of personal protective equipment (PPE), and slaughtering animals on bare floors [8,9,10,11]. Moreover, water used in carcass processing is frequently sourced from untreated supplies, and waste disposal practices are environmentally unsound, posing risks of environmental contamination and foodborne disease outbreaks [12]. In such settings, the synergistic problem between poor animal welfare, inadequate abattoir hygiene, and public health risks are not only profound but cyclic. Poor pre-slaughter animal welfare conditions frequently translate to stress-induced lesions, such as bruises, congestion, haemorrhages, and systemic infections observed during post-mortem inspections [1,2,3]. These lesions are not only indicators of poor welfare but also reduce meat quality through discoloration, increased microbial load, and reduced shelf life [13].

Scientific evidence has repeatedly emphasized the link between compromised animal welfare and increased susceptibility to zoonotic or antimicrobial-resistant pathogens such as *Campylobacter* spp., *Brucella* spp., *Staphylococcus* spp., *Ascaris* spp., cysticerci and *Escherichia coli* O157:H7 [14,15,16,17,18,19,20,21,22,23,24,25,26]. Stress factors such as overcrowding, physical trauma, heat stress, and prolonged transportation are known to suppress immune function in livestock, thereby elevating their pathogen shedding rates and increasing carcass contamination risks during slaughter [9,12]. Post-mortem meat inspection serves as a vital tool in detecting such conditions, yet in many Nigerian abattoirs, inspection is either inadequate or inconsistently conducted due to lack of trained veterinarians or infrastructural support [1,2,9]. The detection of pathognomonic lesions at post-mortem not only signifies poor animal health but also directly impact organ and carcass condemnation rates, with consequent economic and nutritional losses [1].

Carcass handling practices represent a crucial interface between animal welfare failures and human health risk. Poor hygienic practices during carcass processing, evisceration, washing, dressing, and storage, are potent contributors to pathogenic microbial contamination and cross-contamination [13]. These risks are heightened when handlers lack training in food safety principles or neglect essential sanitation protocols such as hand washing, tool sterilization, and environmental cleanliness. Studies have reported high microbial counts, including *E. coli*, *Campylobacter*, and *Staphylococcus*, in Nigerian slaughterhouses, linking these directly to unhygienic processing conditions [14,17,18,19,20,21]. Meat consumed in Enugu State, Nigeria, and other adjoining regions often bypasses thorough post-mortem inspection due to clandestine slaughter and insufficient trained meat inspectors, and such informal supply chains allow contaminated edible animal products to reach consumers, including vulnerable populations such as children, pregnant women, and the immunocompromised [1,9,18,19,20]. The consequences extend beyond gastrointestinal illnesses to include systemic infections and treatment failures, especially where multidrug-resistant bacteria are involved [8,18,26].

Addressing the intricate challenges of poor pre-slaughter animal welfare, prevalent lesions of zoonotic diseases, and unhygienic carcass handling practices in Nigerian slaughterhouses demands a One Health approach that recognizes the interconnection of animal health, human health, and environmental health. While Nigerian government policies such as the Federal Meat Inspection Law (1968) and Animal Disease (Control) Act (2022) provide a regulatory framework, enforcement remains weak at state and local government levels [1,2,9]. There is a pressing need for comprehensive epidemiological data from regions like Enugu State, Nigeria, to inform resource allocation and interventions that can improve slaughterhouse conditions, reduce meat-borne disease risk, and enhance the overall meat value chain. Consequently, the aim of this study is to investigate the pre-slaughter welfare conditions, determine the prevalence of post-mortem lesions of zoonotic and economically important livestock diseases, and carcass handling practices of SHWs in major municipal slaughter facilities in Enugu State, Nigeria, with a focus on their implications for meat quality and public health. Findings from this work will support evidence-based policies and training programmes, targeting abattoir workers, to improve pre-slaughter animal welfare, meat quality, food safety, and public health in Nigeria and other low-and middle-income countries (LMICs).

## 2. Materials and Methods

### 2.1. Ethical Approval and Inform Consent

Ethical clearance for this study was obtained from the Institutional Animal Care and Use Committee (IACUC) of the Faculty of Veterinary Medicine, University of Nigeria, Nsukka, in accordance with established guidelines for the humane use of animals in research (Approval Reference: FVM/UNN/IACUC/2024/078) on 19 January 2024. Oral informed consent was obtained from the leaderships of the slaughterhouse workers’ associations during familiarization visits to the two selected facilities. The leaders approved voluntary participation by interested members and pledged full support for the study. Informed consent from all subjects and/or their legal guardian(s) for publication of identifying information/images in an online open-access publication was obtained prior to the study. Participation was entirely voluntary, with no penalties for non-participation.

### 2.2. The Study Location

The study was conducted in major slaughterhouses (Nsukka, Akwata and 9th mile) located in Enugu State, Southeast, Nigeria. The Nsukka slaughter facility is located in Nsukka while the Akwata and 9th mile slaughterhouses are located in Enugu part of the state. The slaughterhouses were purposively selected given that about 70% of food-producing animals (FPAs) slaughtered in the state are processed in these slaughter facilities. Enugu State, Nigeria, is located on latitude 6°27′10″ N and longitude 7°30′40″ E with an estimated human population of six million. Cattle and pigs are the major FPAs slaughtered and processed in the selected slaughterhouses.

### 2.3. Assessment of the Animal Welfare and Carcass-Processing Practices

The selected slaughter facilities were visited weekly for five months. The research visits were made on Saturdays because the slaughter rates are higher on weekends. Pre-slaughter welfare of cattle was assessed using a customized checklist (Figure S1) adapted from the World Organisation for Animal Health (OIE) Terrestrial Animal Health Code and the Welfare Quality^®^ Assessment Protocol for Cattle [27,28]. Parameters evaluated included evidence of pain, injuries, lameness, body condition, and coat cleanliness. Handling practices such as use of sticks, tail twisting, dragging, and other aversive actions were documented to assess infliction of pain. Behavioural indicators such as slipping, vocalization, and turning back were also recorded. Lairage conditions, including space allowance, water provision, and resting behaviour, were systematically observed to provide a structured appraisal of cattle welfare [27,28].

The transportation conditions, methods of unloading, the state of the lairage facilities, holding conditions, restraint techniques, application of humane handling practices during the slaughter process, were determined by direct observation method [1,2] and documented accordingly. Additionally, the carcass/meat processing practices and method of transporting processed meats to sales outlets were noted and documented. Findings were captured with camera and pictorially presented as figures.

### 2.4. Post-Mortem Carcass Inspection Study

#### 2.4.1. Sample Size Determination

To determine an appropriate sample size for the post-mortem inspection (PMI) study, a minimum of 385 animals for each species surveyed (cattle and pigs) was calculated using the Raosoft^®^ online sample size calculator (http://www.raosoft.com/) accessed on 12 June 2024. This estimation was based on an assumed prevalence of 50%, due to the lack of prior data on the disease lesions in Enugu State, Nigeria. The sample size computation assumed a 5% margin of error, a sample population of 200,000 per species and a 95% confidence level. A total of 1012 cattle and 413 pigs were inspected during the PMI.

#### 2.4.2. Selection of Carcasses for Inspection

All carcasses inspected were randomly selected. A systematic sampling approach was employed, wherein one out of every five carcasses was chosen for examination. The initial carcass from either of the two species per facility was selected using simple random sampling, based on tossing a coin (heads indicating selection). Each selected carcass was then systematically inspected, which included visual examination, palpation, and targeted incisions of key organs and tissues such as the masseter muscle, tongue, lungs, trachea, liver, heart, intercostal muscles, spleen, and kidneys. In addition, lymph nodes, including the retropharyngeal, tracheobronchial, mediastinal, mandibular, parotid, and pre-scapular, were palpated, longitudinally incised, and inspected for pathological lesions.

#### 2.4.3. Post-Mortem Meat Inspection

Post-mortem inspection (PMI) of carcasses and meat, encompassing the head, pluck, organs, and muscles was carried out in accordance with outlined OIE methodologies [27,28]. Visual inspection, palpation, longitudinal incision and olfaction of organs, as deemed appropriate, were made during the carcass inspection to detect pathognomonic gross lesions indicative of prevalent zoonotic or important livestock diseases, including bovine tuberculosis, contagious bovine pleuropneumonia, porcine cysticercosis, contagious caprine pleuropneumonia, dermatophilosis and fascioliasis.

Lungs were inspected for marbling, hepatisation, and pleural adhesions to identify contagious bovine pleuropneumonia. Fasciolosis was diagnosed by palpation and incision of the liver to detect adult flukes in bile ducts. Spotted white disease was identified by gritty, calcified lesions in the liver parenchyma. Liver abscesses were detected through palpation and incision to evaluate encapsulated pus-filled foci. Bovine tuberculosis was diagnosed by inspection and palpation of lungs, lymph nodes, and other organs for caseous granulomas with calcification. Each lesion was documented systematically to determine prevalence.

#### 2.4.4. Data Analyses

Data generated in the study were analyzed using descriptive and inferential statistics using GraphPad Prism^®^ (version 8.0.4, San Diego, CA, USA). The Chi-square test was used to test for simple associations between the occurrence of disease lesions, the outcome variable and explanatory/epidemiological variables (sex, breed, age, location and season). The statistical significance was set at the 5% probability level (significance was accepted at *p* < 0.05).

## 3. Results

### 3.1. Animal Welfare Conditions

The lairage holding the animals prior to slaughter was in poor condition, with no roof and floors flooded when it rained. Cattle waiting for slaughter were kept under the intense heat of the Sun all day long, as there was no roof in the lairage. The animals were crowded due to limited space in some of the slaughterhouses. Slaughter cattle were loaded on trucks and taken to the slaughterhouses. At the lairage, they were dragged down during off-loading (Figure S2). In addition, some of the animals had visible lacerations or bruises, which were likely caused by horn/fighting injuries at the lairage or during transport. The lairage was flooded during the rainy/wet season. Cattle awaiting slaughter were kept on muddy non-concrete floors till the day of slaughter (Figure S2). A pig was observed gasping for air because it was tied to a motorbike at its thoracic/abdominal region (Figure S2). Immobilized slaughter-cattle, likely due to transportation stress or fatigue, were forcefully dragged on lateral recumbent positions from the lairage to the slaughter floor (Figure S3).

Pigs inhumanly transported to the slaughterhouses were thrown down from motorbikes during off-loading. Slaughter-cattle restrained for slaughter (firmly tied fore and hind limbs) were left groaning in excruciating pain for about 30 min before slaughter (Figure S4). Butchers immobilized slaughter-cattle, which were violently struggling due to the painful killing, by stepping on the ventral mandibular region of the animal to reduce aggression (Figure S4). Others were restrained by strangulation/neck twisting to aid bleeding and decapitation, and held in the strangulated position for a while before the slaughter (Figure S5). Animals were bled and decapitated without pre-slaughter stunning.

### 3.2. Carcass-Processing Practices and Slaughterhouse Hygiene

Carcass flaying, evisceration and deboning were performed on slaughterhouse floor where the drainages were water-logged with effluents and breeding maggots (Figure S6). Pig carcasses were dragged on the ground from singeing to the washing points (Figure S6). Heaps of animal dung, oozing out foul smells, and brooding rodents (rats) were seen in the slaughter facilities. Processed carcasses/meats were transported to sale outlets on motorized tricycles and open vans used to convey passengers, cement and wares (Figure S7). At sale outlets, houseflies were observed perching on meat openly displayed for sale on slabs as buyers compared the weights of various meat cuts with their bare hands (Figure S7).

### 3.3. Post-Mortem Inspection Findings

Overall, 45.3% (458/1012) of the slaughter cattle inspected harboured various gross disease related lesions. Lesions of bovine fasciolosis (18%) and CBPP (15.5%) were the most prevalent. Detailed results of the prevalence of all the lesions detected are presented in Table 1. No post-mortem lesion was detected in pig carcasses inspected.

On the temporal distribution, the highest number of lesions was detected in April. The overall monthly distribution of zoonotic and economically important disease lesions detected at post-mortem in cattle slaughtered are shown in Table 2, while the monthly distribution of selected pathological lesions detected is presented in Table 3. There were no significant association (*p* > 0.05) between the occurrence/detection of the lesion and any of the five calendar months (Table 3).

The outcome variable, occurrence of disease lesions was significantly associated (*p* < 0.005) with independent variables—sex, age, breed and slaughterhouse locations (Table 4). The association of various lesions detected and epidemiological variables are shown in Table 5.

## 4. Discussion

### 4.1. Pre-Slaughter Animal Welfare

Poor pre-slaughter animal welfare, characterized by stressful transport and harsh slaughter conditions, as found in this study, is associated with a plethora of biochemical aberrations. Inhumane animal handling disrupts muscle metabolism, leading to the depletion of glycogen reserves essential for post-mortem lactic acid production [29]. Due to the depleted glycogen reserves, such carcasses attain suboptimal post-mortem pH levels of above 5.7, which do not inhibit spoilage bacteria, especially in tropical regions where high ambient temperatures accelerate microbial proliferation [1,29]. These physiological disruptions compromise meat safety and shelf-life, posing direct risks to consumers and contributing to economic losses within the meat industry. Ensuring humane animal handling and proper ante-mortem care is therefore indispensable for maintaining meat quality and preventing post-processing meat spoilage. In addition, poor animal welfare conditions pre-slaughter adversely affect the water-holding capacity (WHC) of meat, a critical determinant of its sensory and physicochemical attributes, including tenderness, juiciness, flavour, and colour [1,30].

The inhibition of lactic acid formation, as a result of depleted muscle glycogen reserve, leads to quality defects like dark, firm, dry (DFD) meat in cattle and pale, soft, exudative (PSE) meat in pigs [30,31]. Stress-induced elevations in creatine phosphokinase (CPK) and aspartate aminotransferase (AST) levels, indicative of tissue damage, subsequently impair meat blooming and diminish overall eating quality. Biochemical changes like protein oxidation and impaired calcium signalling in early post-mortem muscle negatively impact meat quality. These alterations, occurring during the transition from muscle to meat, affect tenderness, WHC, and overall meat palatability [31]. These meat defects not only cause consumer dissatisfaction but also contribute to the loss of a significant portion of the 330 million tonnes of meat produced globally each year [32].

Beyond meat quality, poor pre-slaughter animal welfare significantly affects animal health and public safety. Stressors such as overcrowding, unconducive lairage, prolonged starvation, and the sight of brutality depress immune functions, resulting in lymphoid tissue suppression and low T-cell counts [1,3,29]. Chronic stress leads to elevated cortisol levels, which can suppress immune responses and increase susceptibility to infections in animals. Specifically, prolonged cortisol release can impair T-cell activity, reduce cytokine production, and weaken antibody responses, making animals more vulnerable to both endemic and zoonotic diseases and contributing to chronic inflammatory diseases [4,32,33,34,35]. Meat from such animals, if undercooked or contaminated, may transmit microbial or parasitic pathogens to consumers [36,37,38,39,40,41]. Thus, animal welfare failures at the slaughter stage extend far beyond ethics [42]; they are an integral part of disease prevention and public health safety.

Efforts to promote humane slaughter in Nigeria have faced numerous obstacles, as the meat industry remains dominated by traditional practices and obsolete equipment, compounded by cultural resistance to innovations like pre-slaughter stunning. Although reversible stunning methods, such as head-only electrical stunning, compliant with “halal” requirements, are available and have been shown to enhance bleed-out [43], religious and ideological opposition hinders their widespread adoption. Enhanced bleeding not only minimizes meat contamination with blood-borne pathogens but also improves meat shelf-life by reducing residual haemoglobin and cortisol, both of which catalyze spoilage and lipid oxidation [43,44]. Proper bleed-out reduces the risk of microbial proliferation and oxidative meat degradation, especially in pork, where PSE defects are prevalent.

Addressing the challenges of poor pre-slaughter welfare in Nigeria requires a multidimensional approach. First, the implementation and enforcement of existing frameworks, such as the Strategic Animal Welfare Framework (2016), the Meat Edict of 1988, and the Animal Disease (Control) Act of 2022, must be prioritized. These laws, though present, are often neglected or poorly enforced. Regular training and retraining of slaughterhouse workers in meat hygiene, animal welfare, and food safety are critical. Furthermore, the modernization and mechanization of slaughter facilities, coupled with regulatory oversight, can mitigate the current inhumane pre-slaughter practices. Punitive measures such as fines for inhumane transport and slaughter conditions should be enforced to deter noncompliance and raise revenue for facility improvement. Without these reforms, Nigeria risks continuing a dangerous cycle of poor meat quality, increased foodborne diseases, and public health vulnerabilities in the meat processing value chain.

### 4.2. Carcass-Processing Practices

The poor carcass-/meat-processing practices observed raise doubts on the safety of meats produced and further aggravate the possibilities of inter- and intra-species transmission of infections, particularly meat-borne zoonoses in the study area. Dressing carcasses/meats on bare slaughterhouse floors, use of water of unproven microbial quality for meat processing [15], and non-use of PPE during slaughterhouse operations pose great public health risks to meat consumers and processors [10,15]. However, these findings are not entirely surprising considering that some slaughterhouse workers in Nigeria are not formally trained in carcass/meat processing [1,24]. Slaughterhouses in Nigeria frequently operate under substandard conditions, falling short of internationally recognized hygiene and safety benchmarks. Deficiencies are commonly observed in infrastructural adequacy, sanitary waste disposal systems, and compliance with systematic ante-mortem and post-mortem meat inspection protocols, thereby posing significant risks to both food safety and public health [36,37,45].

The meat-handling practices observed across the slaughter facilities and meat sales outlets present critical food safety breaches and profound public health threats. The practice of flaying, eviscerating, and deboning carcasses on bare slaughterhouse floors contaminated with effluent and breeding maggots constitutes a major hazard for microbial contamination, as processed meat can easily become contaminated with harmful bacteria, potentially leading to foodborne illnesses [24]. These unsanitary conditions of the slaughterhouse environment encourage the proliferation of zoonotic and enteric pathogens and their vectors [46]. The dragging of pig carcasses across dirty floors, exposure to animal dung, and infestation by rodents, which are notorious reservoirs for *Leptospira* spp., *Yersinia pestis*, and *Campylobacter* spp., further increase the risk of contamination, cross-infection, and disease outbreaks [47].

Such unhygienic slaughterhouse environments, which are common in abattoirs in Nigeria and other developing countries, could lead to increased microbial load on meat surfaces [48,49]. These conditions violate both national and international meat hygiene codes, which mandate clean, pest-free environments, proper drainage, and carcass handling on sanitary surfaces. Furthermore, the transportation of meat in open vans and motorized tricycles previously used for conveying cement and passengers constitutes a clear breach of hygienic standards, likely resulting in chemical and microbial contamination. Similar risky transportation practices were documented in South Africa, where improper meat handling post-processing contributed significantly to *Staphylococcus* spp. and coliform contamination on meat [50].

At the retail level, the presence of houseflies perching on exposed meat surfaces and the direct handling of meat by buyers with unwashed hands represent additional vectors for microbial transfer. Houseflies are proven mechanical carriers of many pathogens, including *Shigella* spp., *Campylobacter* spp., and *Salmonella* spp. [51]. Open-air meat display without temperature control or protective barriers exacerbates spoilage, increases the risk of toxin production by psychrotrophic bacteria, and promotes the spread of antimicrobial-resistant organisms, especially in hot tropical climates [52]. These poor hygiene practices, when sustained, contribute to the burden of foodborne diseases and even zoonotic outbreaks, a serious concern for both public health and veterinary services under the One Health framework.

### 4.3. Post-Mortem Inspection

The detection of multiple zoonotic and economically significant disease lesions in slaughtered cattle in Enugu State has profound public health implications. The high prevalence of contagious bovine pleuropneumonia (15.5%), bovine fasciolosis (18%), liver abscesses (6.6%), and ascariasis (4.6%) suggests ongoing endemicity of infectious and parasitic diseases in the cattle population. Bovine tuberculosis, although less prevalent (0.5%), is of particular concern due to its zoonotic potential that results in chronic respiratory illness in humans, especially among abattoir workers, meat handlers, and consumers exposed to undercooked or contaminated meat [53,54,55].

At the national level, the observed prevalence of bovine tuberculosis at 0.5% is markedly lower than the 2.9% previously reported across various states in Nigeria [40] and the 7% recorded in Lagos State, Nigeria [52]. At the international level, this prevalence is also lower than the 21.7% reported in Cameroon [56] and the 39.3% documented in Ethiopia [57]. The discrepancies in prevalence rates may be attributable to differences in the sensitivity and specificity of diagnostic techniques, as well as ecological and epidemiological variations such as the extent of cattle–wildlife interaction, which constitutes a recognized transmission pathway for *Mycobacterium bovis* [55,58]. The comparatively low prevalence reported notwithstanding, sustained and coordinated efforts toward the control and eventual eradication of bovine tuberculosis remain imperative, given its zoonotic potential, public health significance, and implications for food safety and global One Health security.

Monthly distribution data indicate notable seasonal patterns, with higher infection rates in February to April (above 50% prevalence) compared to May (37.7%), suggesting that climatic factors such as rainfall onset and grazing patterns may influence pathogen transmission. This is consistent with earlier observations that wet seasons favour the survival of fasciola intermediate hosts and facilitate respiratory pathogen spread among cattle in communal grazing systems [59]. Epidemiological analysis further reveals significantly higher lesion prevalence in females (66.7%) compared to males (44.1%), possibly due to prolonged retention of breeding females in herds, increasing cumulative exposure to infectious agents. Age-related patterns, with younger cattle (<4 years) showing higher prevalence (54.1%) than older ones (45.4%), could reflect immature immunity or stress-related susceptibility in younger animals. Breed predisposition, with White Fulani cattle exhibiting the highest lesion rates, may be influenced by breed-specific management and movement patterns, as this breed is predominant in transhumant pastoral systems in Nigeria [60].

From a public health standpoint, these findings emphasize the dual burden of zoonoses and food safety concerns. The detection of tuberculosis and CBPP lesions raises the possibility of unrecognized human cases linked to exposure through raw or undercooked meat and unpasteurized milk, while fasciolosis and hepatic abscesses highlight the potential for chemical and microbial contamination of meat products. Previous studies [11,31] linked such abattoir-detected lesions to high-risk meat supply chains and inadequate post-slaughter meat inspection enforcement, which creates a silent transmission pathway for zoonotic pathogens, particularly in informal markets where inspection standards are poorly regulated. Beyond human health, these diseases could impose significant economic losses through carcass condemnation, reduced productivity, and increased veterinary costs, further undermining food security and livelihoods.

### 4.4. Limitation of the Study

Diagnostic reliance on gross post-mortem lesions without molecular confirmation, may underestimate subclinical infections. Despite this limitation, the findings provide valuable insights into the interplay between pre-slaughter welfare, carcass hygiene, and zoonotic disease risk, informing targeted interventions for meat safety and public health security in Nigeria and other LMICs.

## 5. Conclusions

This study identified critical deficiencies in Nigerian municipal slaughterhouses. Major gaps were observed in pre-slaughter animal welfare, carcass-processing practices, and post-mortem inspection, with serious implications for meat quality, food safety, and public health. The high prevalence of pathological lesions in cattle, particularly fasciolosis (18%) and contagious bovine pleuropneumonia (15.5%), underscores the endemicity of economically important and zoonotic diseases. Poor hygiene, unsanitary meat processing, and inhumane handling practices further reveal systemic regulatory and infrastructural shortcomings. Variations in lesion occurrence across demographic and environmental factors highlight persistent epidemiological risks within the livestock value chain. Strengthening ante- and post-mortem inspection systems, mechanizing slaughter operations, and enforcing animal welfare and meat hygiene regulations are imperative. Implementing targeted disease control measures, including vaccination and deworming, will further reduce disease burden. Continuous training of abattoir workers and modernization of facilities, supported by robust surveillance within a One Health framework, are essential for safeguarding public health and ensuring sustainable meat production in Nigeria.

## Figures and Tables

**Table 1:** Prevalence of lesions associated with zoonotic and economically important disease that were detected during post-mortem inspection of cattle carcass (*n* = 1012) slaughtered for human consumption in Enugu State, Nigeria.

S/n	Disease Lesions	Number (%) of Carcasses with Lesion	Number (%) of Carcasses Without Lesions	χ^2^-Value	*p*-Value
1.	CBPP	157 (15.5)	855 (84.5)	271	0.012 *
2.	Bovine tuberculosis	5 (0.5)	1007 (99.5)		
3.	Bovine fasciolosis	182 (18)	830 (82)		
4.	Ascariasis	47 (4.6)	965 (95.4)		
5.	Liver abscess	67 (6.6)	945 (93.4)		

CBPP = contagious bovine pleuro-pneumonia, * = statistically significant *p*-value, Chi-square statistics (GraphPad Prism^®^ software, version 8.0.4, San Diego, CA, USA).

**Table 2:** Overall monthly distribution of zoonotic and economically important disease lesions detected at post-mortem in cattle slaughtered for human consumption in Enugu State, Nigeria.

Months	Number of Cattle Inspected	Number of Infected Cattle (%)	Number of Uninfected Cattle (%)	χ^2^-Value	*p*-Value
February	72	37 (51.4)	35 (48.6)	30	0.021 *
March	88	45 (51.2)	43 (48.8)		
April	82	56 (68.3)	26 (31.7)		
May	435	164 (37.7)	272 (62.3)		
June	335	156 (46.6)	179 (53.4)		

* = Statistically significant *p*-value; Chi square test (GraphPad Prism^®^ software, version 8.0.4, San Diego, CA, USA).

**Table 3:** Monthly distribution of selected pathological lesions detected in cattle slaughtered in Enugu State, Nigeria.

Lesion	Month	Number of Cattle Examined	Number of Cattle with Lesion (%)	Number of Cattle Without Lesion (%)	χ^2^-Value	*p*-Value
CBPP	February	72	13 (18.1)	59 (81.9)	6.8	0.1441
	March	88	16 (18.2)	72 (81.8)		
	April	82	19 (23.2)	63 (76.8)		
	May	435	56 (12.9)	379 (87.1)		
	June	335	53 (15.8)	282 (84.2)		
Bovine fasciolosis	February	72	15 (20.8)	57 (79.2)		
	March	88	17 (19.3)	71 (80.7)	8.2	0.0843
	April	82	22 (26.8)	60 (73.2)		
	May	435	65 (14.9)	370 (85.1)		
	June	335	62 (18.5)	273 (81.5)		
Ascariasis	February	72	4 (5.6)	68 (94.4)	2.2	0.7079
	March	88	5 (5.7)	83 (94.3)		
	April	82	6 (7.3)	76 (92.7)		
	May	435	17 (3.9)	418 (96.1)		
	June	335	16 (4.8)	319 (95.2)		
Liver abscess	February	72	5 (6.9)	67 (93.1)	2.5	0.6521
	March	88	7 (8)	81 (92)		
	April	82	8 (9.8)	74 (90.2)		
	May	435	24 (5.5)	411 (94.5)		
	June	335	23 (6.9)	312 (93.1)		

CBPP = contagious bovine pleuro-pneumonia, Chi square test (GraphPad Prism^®^ software, version 8.0.4, San Diego, CA, USA).

**Table 4:** Distribution of lesions of zoonotic and economically important disease in cattle slaughtered for human consumption in Enugu State by the various epidemiological factors.

Epidemiological Variable	Levels	Numbers of Cattle Inspected	Number of Cattle with Lesions (%)	Number of Cattle Without Lesions (%)	*p*-Value
Sex	Male	961	424 (44.1)	537 (55.9)	0.0011 *
	Female	51	34 (66.7)	17 (33.3)	
Breed	White Fulani	982	447 (45.5)	534 (54.5)	0.0013 *
	Other breeds	30	11 (36.7)	19 (63.3)	
Age	<4 years	455	205 (54.1)	250 (54.9)	0.0014 *
	≥4 years	557	253 (45.4)	304 (54.6)	
Location	Enugu	152	69 (45.4)	83 (54.6)	0.0021 *
	Nsukka	860	389 (45.2)	471 (54.8)	
Season	Rainy/wet	770	348 (45.2)	422 (54.8)	0.0028 *
	Dry/hot	242	110 (45.5)	132 (54.5)	

* = Statistically significant *p*-value; Chi square test (GraphPad Prism^®^ software, version 8.0.4, San Diego, CA, USA).

**Table 5:** Association of various epidemiological variables with occurrence of selected pathological lesions detected in cattle slaughtered in Enugu State, Nigeria.

Epidemiological Variable	Category	Number of Cattle with Lesions (%)	Number of Cattle Without Lesions (%)	χ^2^-Value	*p*-Value
Contagious bovine pleuro-pneumonia (*n* = 157)					
Sex	Male	140 (14.6)	816 (85.4)	13.4	0.0034 *
	Female	17 (33.3)	39 (66.6)		
Breed	White Fulani	153 (15.6)	829 (84.4)	0.11	0.7371
	Other breeds	4 (13.3)	26 (86.7)		
Age	<4 years	50 (11)	405 (89)	12.9	0.0014 *
	≥4 years	107 (19.2)	450 (80.8)		
Location	Enugu	24 (15.8)	128 (84.2)	0.11	0.9189
	Nsukka	133 (15.5)	727 (84.5)		
Season	Rainy/wet	119 (15.5)	651 (84.5)	0.11	0.9261
	Dry/hot	38 (15.7)	204 (84.3)		
Bovine fasciolosis (*n* = 182)					
Sex	Male	168 (17.5)	793 (82.5)	3.3	0.0709
	Female	14 (27.5)	37 (72.5)		
Breed	White Fulani	178 (18.1)	804 (81.9)	0.45	0.5007
	Other breeds	4 (13.3)	26 (86.7)		
Age	<4 years	62 (13.6)	393 (86.4)	11	0.0011 *
	≥4 years	120 (21.5)	437 (78.5)		
Location	Enugu	27 (17.8)	125 (82.2)	0.06	0.9386
	Nsukka	155 (18)	705 (82)		
Season	Rainy/wet	158 (20.5)	612 (79.5)	14	0.0002 *
	Dry/hot	24 (9.9)	218 (90.1)		
Ascariasis (*n* = 47)					
Sex	Male	40 (4.2)	921 (95.8)	18	0.0001 *
	Female	9 (17)	44 (83)		
Breed	White Fulani	43 (8)	939 (92)	1.1	0.3051
	Other breeds	4 (13.3)	26 (86.7)		
Age	<4 years	31 (6.8)	424 (93.2)	8.8	0.0034 *
	≥4 years	16 (2.9)	541 (93.1)		
Location	Enugu	7 (4.6)	145 (95.4)	0.01	0.9801
	Nsukka	40 (4.7)	820 (95.3)		
Season	Rainy/wet	41 (5.3)	729 (94.7)	3.4	0.0666
	Dry/hot	6 (2.5)	236 (97.5)		
Liver abscess (*n* = 67)					
Sex	Male	62 (6.5)	899 (93.5)	0.88	0.3481
	Female	5 (9.8)	46 (90.2)		
Breed	White Fulani	62 (6.3)	920 (96.7)	5	0.0247 *
	Other breeds	5 (16.7)	25 (83.3)		
Age	<4 years	37 (8.1)	418 (91.9)	3.1	0.0805
	≥4 years	30 (5.4)	527 (94.6)		
Location	Enugu	15 (9.9)	137 (90.1)	3.1	0.0806
	Nsukka	52 (6)	808 (94)		
Season	Rainy/wet	51 (6.6)	719 (93.4)	0.68	0.4079
	Dry/hot	16 (6.6)	226 (93.4)		

* = Statistically significant *p*-value; Chi square test (GraphPad Prism^®^ software, version 8.0.4, San Diego, CA, USA).

## Data Availability

The original contributions presented in this study are included in the article/Supplementary Material. Further inquiries can be directed to the corresponding author.

## Supplementary Materials

The following supporting information can be downloaded at https://www.mdpi.com/article/10.3390/vetsci13050439/s1, Figure S1: Pre-Slaughter Animal Welfare Assessment Checklist for Cattle; Figure S2: Cattle dragged during off-loading, cattle held in deep muddy floors, and a pig tied on a motorbike showing respiratory distress en route to slaughter; Figure S3: Immobilized or lame slaughter-cattle being dragged forcefully from the lairage to the slaughter floor at one of the slaughterhouses in Southeast Nigeria; Figure S4: Slaughter practices involving severe distress: cattle restrained by tightly binding fore and hind limbs and left in pain prior to bleeding; and a butcher forcefully immobilising a struggling animal by neck twisting and stepping on the ventral mandibular region; Figure S5: Slaughter cattle restrained by strangulation/neck twisting to aid bleeding and decapitation. The animals are sometimes held in the strangulated position for five to 10 min before the slaughter; Figure S6: Unsanitary slaughter conditions: bovine carcasses flayed and dressed amid effluent-flooded, maggot-infested drains; and singed pig carcasses dragged along the ground to the washing point; Figure S7: Post-processing hygiene risks: carcasses transported by tricycle and open vans; and meat openly displayed at outlets with fly contact and handled by buyers with bare hands.

## Abbreviations

The following abbreviations are used in this manuscript:

FPAs	Food-producing Animals
OIE	World Organisation for Animal Health
PMI	Post-mortem Inspection
CBPP	Contagious Bovine Pleuro-Pneumonia
IACUC	Institutional Animal Care and Use Committee (IACUC)
WHC	Water holding capacity
DFD	Dark, Firm and Dry meat
CPK	Creatinine Phosphokinase
AST	Aspartate Aminotransferase
PSE	Pale, Soft, Exudative meat
