# Supplementary material for: Animal Welfare, Carcass-Processing Practices and Post-Mortem Lesions in Nigerian Municipal Slaughterhouses: Implications for Meat Quality and Public Health Security
Source: Vet Sci. 2026 Apr 30;13(5):439. doi: 10.3390/vetsci13050439 (PMC13211438; doi:10.3390/vetsci13050439)
Supplement: Supplementary file 1 [file vetsci-13-00439-s001.zip › vetsci-4239685-supplementary.pdf]

Figure S1: **Customized Pre-Slaughter Animal Welfare Assessment Checklist for Cattle**

This checklist is adapted from the World Organisation for Animal Health (OIE) Terrestrial Animal Health Code (Chapter 7.5 – Slaughter of animals) and the Welfare Quality® Assessment Protocol for Cattle (2009). It is designed for use in municipal slaughterhouses in Nigeria to evaluate pre-slaughter animal welfare, with emphasis on handling practices, infliction of pain, and lairage conditions.

1. Animal Health and Physical Condition

- ☐ Body condition score (Good / Fair / Poor)
- ☐ Presence of visible injuries or wounds (Yes / No)
- ☐ Signs of lameness (None / Mild / Severe)
- ☐ Coat cleanliness (Clean / Moderately dirty / Very dirty)

2. Handling Practices

- ☐ Use of sticks (Yes / No)
- ☐ Tail twisting (Yes / No)
- ☐ Dragging of animals (Yes / No)
- ☐ Use of electrical prods or other aversive methods (Yes / No)
- ☐ Frequency of vocalization during handling (None / Occasional / Frequent)

3. Behavioral Indicators of Distress

- ☐ Slipping or falling during movement (None / Occasional / Frequent)
- ☐ Turning back or refusing to move (Yes / No)
- ☐ Excessive vocalization (Yes / No)
- ☐ Signs of fear or agitation (Calm / Moderate / Severe)

4. Lairage Assessment

- ☐ Space allowance (Adequate / Inadequate)
- ☐ Provision of water (Available / Not available)
- ☐ Resting behavior observed (Yes / No)
- ☐ Overcrowding (Yes / No)
- ☐ Environmental conditions (Well-ventilated / Poor ventilation)

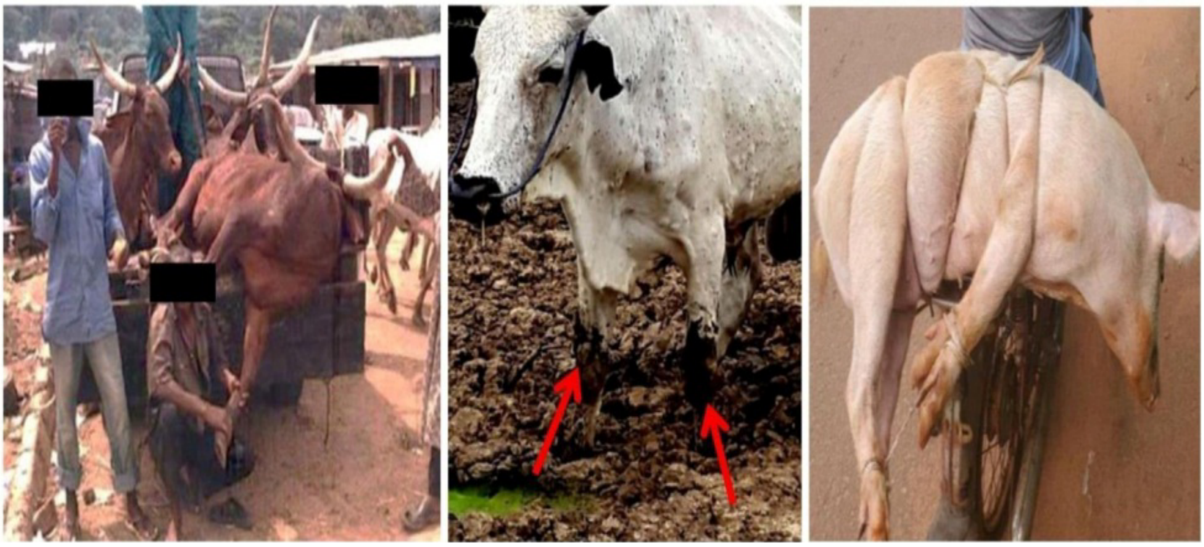

Figure S2: Cattle dragged during off-loading, cattle held in deep muddy floors, and a pig tied on a motorbike showing respiratory distress en route to slaughter.

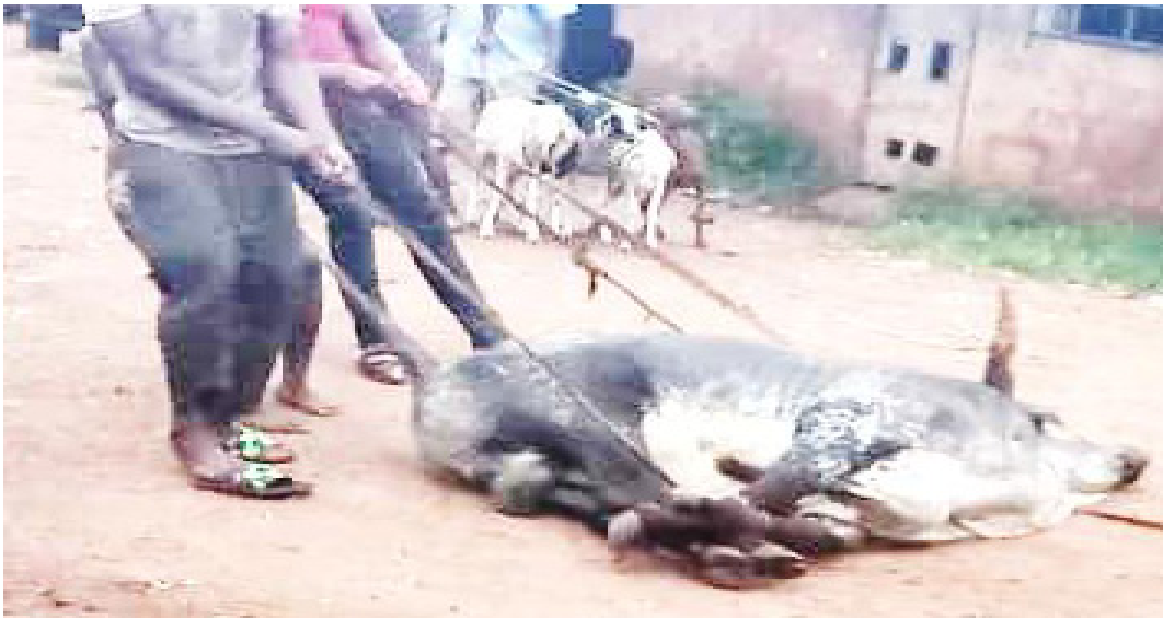

Figure S3: Immobilized or lame slaughter-cattle being dragged forcefully from the lairage to the slaughter floor at one of the slaughterhouses in Southeast Nigeria

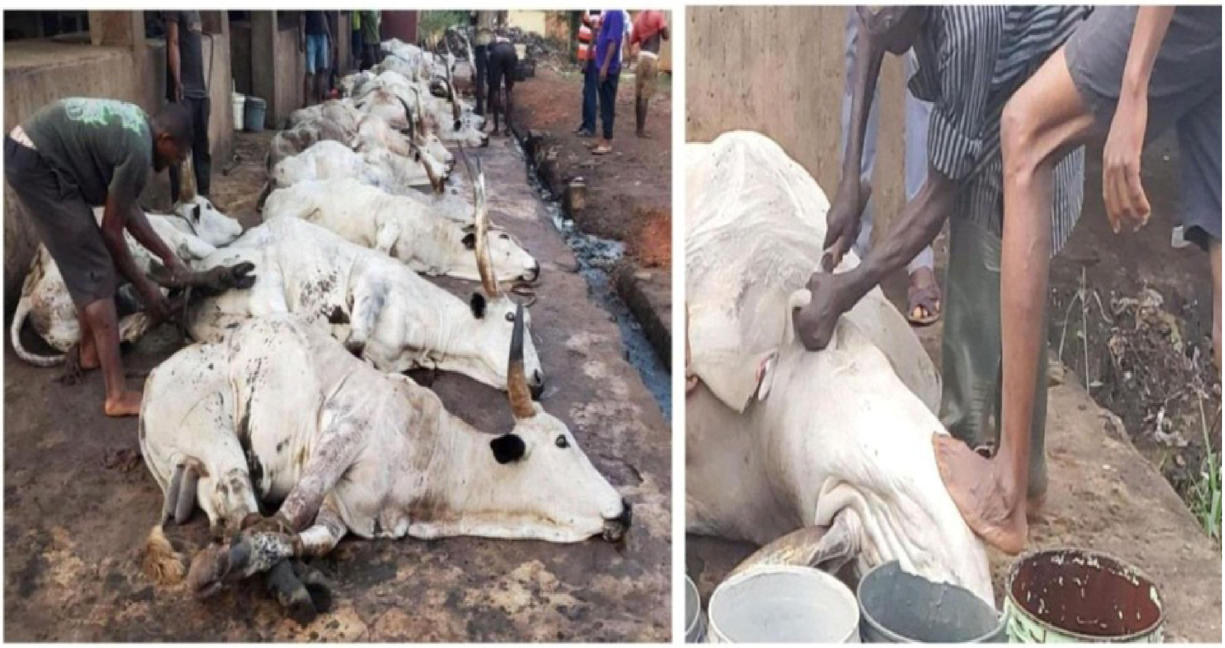

Figure S4: Slaughter practices involving severe distress: cattle restrained by tightly binding fore and hind limbs and left in pain prior to bleeding; and a butcher forcefully immobilising a struggling animal by neck twisting and stepping on the ventral mandibular region

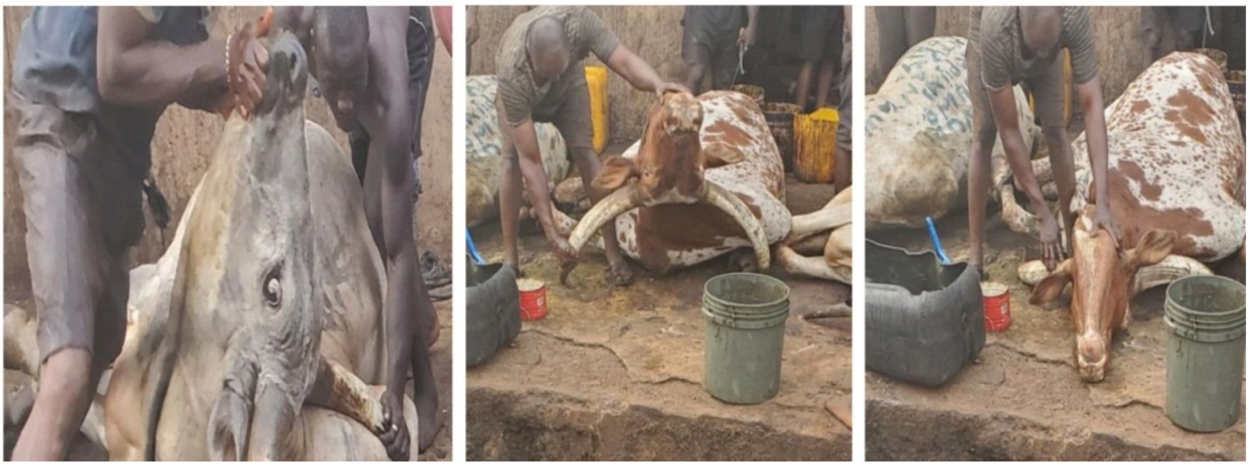

Figure S5: Slaughter cattle restrained by strangulation/neck twisting to aid bleeding and decapitation. The animals are sometimes held in the strangulated position for five to 10 minutes before the slaughter.

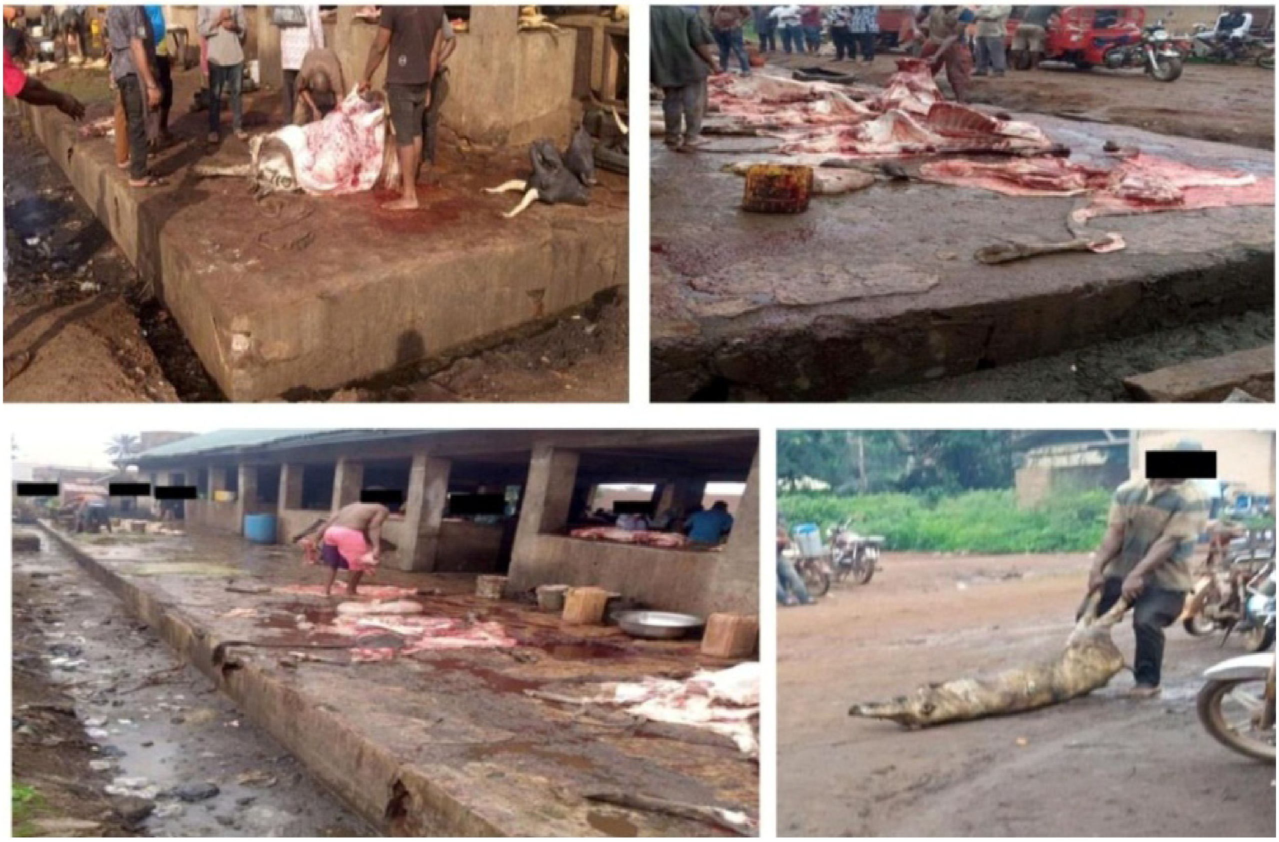

Figure S6: Unsanitary slaughter conditions: bovine carcasses flayed and dressed amid effluent-flooded, maggot-infested drains; and singed pig carcasses dragged along the ground to the washing point.

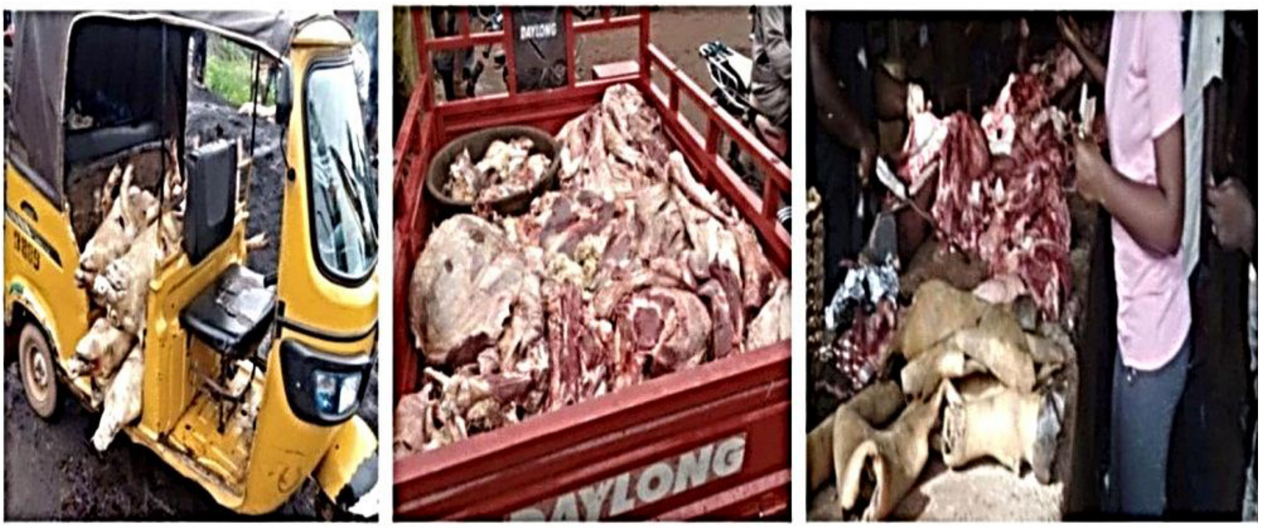

Figure S7: Post-processing hygiene risks: carcasses transported by tricycle and open vans; and meat openly displayed at outlets with fly contact and handled by buyers with bare hands.
